# Closing Editorial: Immunopathogenesis of Bacterial Infection

**DOI:** 10.3390/cells14231894

**Published:** 2025-11-28

**Authors:** Sasha Shafikhani

**Affiliations:** Department of Dermatology, School of Medicine, University of California Davis, Sacramento, CA 95817, USA; sshafikhani@health.ucdavis.edu

## 1. Summary of the Special Issue

Bacterial infections remain a persistent and evolving threat to human health worldwide. Beyond the continuing challenge of antibiotic resistance, it is increasingly clear that the outcomes of bacterial disease are dictated by the complex interplay between pathogen virulence, host immune responses, tissue-specific microenvironments, and temporal dynamics of infection. Recognizing this, this Special Issue of *Cells* titled “Immunopathogenesis of Bacterial Infection” was launched to address these critical gaps and advance our mechanistic insights, bringing together recent advances in bacterial pathogenesis, host immunology, and the emerging paradigm of host-directed therapies. A total of ten papers were published in this Special Issue, collectively garnering 399 citations to date as assessed on Google Scholar within just two years, highlighting its success.

## 2. What has Changed Since?

Four key advances stand out.

(1) **Immunometabolism as a driver (not a passenger) of pathogenesis**. A growing body of research has revealed the bidirectional interplay between host metabolism and antibacterial immunity. In the gut–liver axis, for example, enteric infection was shown to remodel bile metabolites (including itaconate) in ways that tune innate defenses and microbial ecology [1]. Macrophage activation programs are now being mechanistically linked to microbial metabolites and cellular energy flux, reframing our understanding of “nutrient” signals as integral regulators of host defense during infection [2].
(2) **Tissue education and trained immunity beyond macrophages.** The concept of trained innate immunity has broadened beyond monocytes/macrophages to encompass additional cell types and tissue compartments. A recent elegant study reveals that a localized *Staphylococcus aureus* skin infection can imprint an eosinophil-based “innate immune memory,” exerting systemic effects on subsequent allergic inflammation—a striking demonstration of organ-level crosstalk that reshapes later immune responses to unrelated stimuli [3]. Complementary reviews suggest that, when properly directed, trained immunity could reduce susceptibility to diverse bacterial infections while minimizing the risk of chronic inflammatory sequelae [4].
(3) **Antibiotic action and resistance as immunological phenomena.** Antibiotic resistance remains a constantly shifting target: recent studies demonstrate that ESKAPE pathogens can acquire resistance to even “next-generation” antibiotics within weeks in vitro, with corresponding mutations already detectable in clinical isolates and environmental microbiomes [5]. This sobering observation highlights that stewardship alone is insufficient to curb resistance. Rather, effective strategies must incorporate host immunobiology. In parallel, antibiotic therapy itself can elicit unintended immune consequences. For example, bactericidal antibiotics have been shown to trigger exaggerated and damaging cytokine responses through TLR9 sensing of bacterial DNA, underscoring the need to tailor antibiotic class or combination choices for patients at heightened risk of immunopathology and tissue injury [6].
(4) **Immunomodulator to the rescue**. In recent years, researchers have increasingly focused on immunomodulatory strategies that enhance or redirect host responses. In surgical and implant-associated infections, administration of immunomodulators, such as N-formyl-methionyl-leucyl-phenylalanine (fMLP) has been shown to stimulate neutrophil recruitment and reduce infection burden as effectively as a powerful systemic antibiotic against *Pseudomonas aeruginosa* and *Staphylococcus aureus* [7,8]. Similarly, immunomodulators that restore neutrophil function in diabetic hosts or reprogram macrophage polarization toward a bactericidal phenotype have been effective against infection and improved healing [9,10]. Collectively, these studies represent a shift from pathogen-centric to host-centric intervention strategies.

## 3. From Mechanisms to Levers

A welcome trend is the emergence of host-directed adjuvants that “change the battlefield” rather than only the bullet. Nature Microbiology recently reported a small-molecule host adjuvant that rescues antibiotic killing of intracellular persisters by re-energizing bacteria within macrophages while dampening host-derived ROS/RNS that drive antibiotic tolerance. This metabolic “nudge” collapses dormancy and sensitizes pathogens, including *S. aureus* and *Salmonella*, to conventional drugs in vivo [11]. It is difficult to overstate the conceptual significance of this finding that antibiotic tolerance is not solely a bacterial adaptation but a host-conditioned state that can, in principle, be therapeutically manipulated.

Pathogenesis is also an ecological problem. Sophisticated contact-dependent antagonism within the microbiota sculpts community structure and, indirectly, mucosal immunity—reminding us that interbacterial warfare affects the host’s inflammatory set-point and colonization resistance [12]. On another frontier, phage–bacteria arms races reveal how phages evade cognate bacterial immune systems, influencing bacterial community dynamics and, by extension, host immune exposures [13]. Even when they appear to concern bacteria alone, these interactions resonate throughout host immune pathways.

## 4. Priorities, Platforms, and Patient Impact

Antimicrobial resistance (AMR) and immunopathology remain priorities. The 2024 World Health Organization (WHO) bacterial priority pathogen list, which elevates several Gram-negative organisms and *Mycobacterium tuberculosis* [14], should be considered alongside the cellular immunology studies highlighted in this Special Issue. The priority pathogens are precisely those that manipulate innate signaling, subvert phagocyte effector programs, and exploit host metabolic niches. For example, *Pseudomonas aeruginosa* deploys the conserved toxin ExoT to disrupt NLRC4 inflammasome-mediated host immunity during infection [15]. Target selection in drug discovery and vaccine design must pivot away from antibiotics and toward immunomodulators that can overcome bacterial virulence factors while mobilizing host immunity against infection.

## 5. Methodological Platforms Enabling New and Clinically Relevant Insights

Single-cell and spatial atlases of infected tissues now parse neutrophil and macrophage states associated with severe bacterial pneumonia, including immature neutrophil programs linked to tissue injury [16]. In addition, an elegant 14-year longitudinal in-host evolution study shows how *Yersinia enterocolitica* adapt under immune and antibiotic pressure, often trading growth for persistence to ensure survival [17]. Clinical translation benefits when we integrate these layers.

## 6. Where We Go Next

Drawing on the articles in this Special Issue and recent advances across high-impact journals, we see five practical directions:

1. Exploit organ–organ immune circuits. The gut–liver axis is not merely collateral damage during enteric infection; it is a programmable circuit. Mapping metabolite flows (e.g., itaconate and related dicarboxylates) and their cellular targets can reveal levers to enhance mucosal defense without provoking collateral inflammation [1].
2. Design regimens around tolerance, not just resistance. If host ROS/RNS in macrophages potentiate antibiotic tolerance, then rational combinations might pair an antibiotic with a host-directed adjuvant that tempers those signals—precisely the KL1 logic emerging from recent work. Prospective clinical studies should track tolerance biomarkers (metabolic and transcriptional) alongside MICs [11].
3. Choose antibiotics with the immune system in mind. For patients at high risk of immunopathology, bacteriostatic or DNA-sparing regimens—or the addition of targeted anti-inflammatory co-therapies—could reduce TLR9-driven cytokine storms observed with some bactericidal regimens, without sacrificing clearance. Translational trials should incorporate immune readouts as endpoints [6].
4. Invest in immunomodulator-based strategies to combat infection. Considering the staggering rate of AMR, it is now important more than ever to seek approaches that can overcome pathogens’ stealth mechanisms [15] and mobilize host innate immune responses to combat infection [9].
5. Leverage microbial competition. Harnessing contact-dependent inhibition and other antagonistic interactions may offer microbiota-level strategies that suppress pathogens while stabilizing host-beneficial communities, decreasing the inflammatory “tone” that predisposes to pathology [12].
6. Anticipate evolution—clinically. Routine sequencing of persistent infections (including biofilm-associated disease) and adaptive dosing guided by evolutionary risk landscapes can discourage resistance trajectories documented across ESKAPE organisms. Collaboration between evolutionary microbiologists and clinicians is no longer optional [5].

## 7. Gratitude and Community

A Special Issue stands on the labor of a community. We thank the authors for submitting rigorous, creative work and our reviewers for their thoughtful assessments under tight timelines. We appreciate the editorial staff at *Cells* for maintaining a fast yet careful process that prizes scientific soundness and transparency.

## Data Availability

No new data were created or analyzed in this study. Data sharing is not applicable to this article.
